# Clinical recognition of acute aortic dissections: insights from a large single-centre cohort study

**DOI:** 10.1007/s12471-016-0921-8

**Published:** 2016-11-23

**Authors:** W. W. Jansen Klomp, G. J. Brandon Bravo Bruinsma, L. M. Peelen, A. P. Nierich, J. G. Grandjean, A.W.J. van ’t Hof

**Affiliations:** 1 Department of Cardiology, Isala, Zwolle, The Netherlands; 2 Department of Clinical Epidemiology, Julius Center for Health Sciences and Primary Care, University Medical Center Utrecht, Utrecht, The Netherlands; 3 Department of Cardiothoracic Surgery, Isala, Zwolle, The Netherlands; 4 Department of Anaesthesiology, University Medical Center Utrecht, Utrecht, The Netherlands; 5 Department of (Thoracic) Anaesthesia and Intensive Care, Isala, Zwolle, The Netherlands; 6 MIRA Institute for Biomedical Technology and Technical Medicine, University of Twente, Enschede, The Netherlands

**Keywords:** Aortic dissection, Back pain, Clinical suspicion, Differential diagnosis, Female

## Abstract

**Aims:**

Acute aortic dissection (AD) requires immediate treatment, but is a diagnostic challenge. We studied how often AD was missed initially, which patients were more likely to be missed and how this influenced patient management and outcomes.

**Methods:**

A retrospective cohort study including 200 consecutive patients with AD as the final diagnosis, admitted to a tertiary hospital between 1998 and 2008. The first differential diagnosis was identified and patients with and without AD included were compared. Characteristics associated with a lower level of suspicion were identified using multivariable logistic regression, and Cox regression was used for survival analyses. Missing data were imputed.

**Results:**

Mean age was 63 years, 39% were female and 76% had Stanford type A dissection. In 69% of patients, AD was included in the first differential diagnosis; this was less likely in women (adjusted relative risk [aRR]: 0.66, 95% CI: 0.44–0.99), in the absence of back pain (aRR: 0.51, 95% CI: 0.30–0.84), and in patients with extracardiac atherosclerosis (aRR: 0.64, 95% CI: 0.43–0.96). Absence of AD in the differential diagnosis was associated with the use of more imaging tests (1.8 vs. 2.3, *p* = 0.01) and increased time from admission to surgery (1.8 vs. 10.1 h, *p* < 0.01), but not with a difference in the adjusted long-term all-cause mortality (hazard ratio: 0.76, 95% CI: 0.46–1.27).

**Conclusion:**

Acute aortic dissection was initially not suspected in almost one-third of patients, this was more likely in women, in the absence of back pain and in patients with extracardiac atherosclerosis. Although the number of imaging tests was higher and time to surgery longer, patient outcomes were similar in both groups.

**Electronic supplementary material:**

The online version of this article (doi:10.1007/s12471-016-0921-8) contains supplementary material, which is available to authorized users.

## Introduction

Acute aortic dissection (AD) is a vascular emergency that requires immediate treatment to prevent morbidity and mortality. However, its diagnosis is challenging given heterogeneous symptoms and a low incidence estimated at 2.6–6 cases per 100,000 person-years [1–3]. Although 8–10% of all patients admitted to a general emergency care department present with acute chest pain, only one in 980 of these patients will have an acute aortic dissection [4, 5].

The mortality of untreated AD has been estimated to be 1% per hour [1]; a prompt diagnosis is thus paramount. Despite the diagnostic tests for aortic dissections being highly sensitive, dissections are frequently missed due to insufficient recognition of its symptoms. It has been estimated that only half of all patients with AD are admitted to a hospital [3], and 23% of patients with Stanford type A dissections are not identified until surgery for another indication or necropsy [6]. One of the steps to improve the recognition of future AD patients is to identify the characteristics associated with a higher likelihood of a missed diagnosis.

We aimed to study the clinical awareness of AD during the initial work-up of patients in whom AD was the final diagnosis. Since it is by definition difficult to identify patients with a missed diagnosis, we used the first documented differential diagnosis to identify patients in whom AD was not suspected initially. Absence of AD in the differential diagnosis indicates that the symptoms were not attributed to acute AD as the underlying condition. We studied in what proportion of patients AD was not recognised initially. Secondly, we identified the characteristics which were associated with lower clinical recognition and, thirdly, we evaluated the impact on patient management and outcome.

## Methods

### Study design

This retrospective cohort study included all consecutive patients with AD as their final diagnosis, admitted to a large tertiary hospital (Isala, Zwolle, Netherlands) between January 1998 and June 2008. Patients with a traumatic, iatrogenic or chronic dissection were excluded as were patients aged less than 18 years and patients referred for recovery after the diagnosis and treatment of AD in another hospital. Our institution’s ethics committee waived formal evaluation of the study, which conformed to the principles outlined in the Helsinki declaration.

### Patient identification and data collection

Surgically treated patients were identified from a prospective surgical registry, which included patient history, perioperative data, and follow-up of in-hospital complications [7]. Patients treated medically were identified retrospectively from a central electronic database containing all hospital discharge diagnoses. We used the search term ‘dissection’, which was purposely kept broad in order to capture all AD patients. The final diagnosis was established after review of the electronic discharge letter, or if this did not suffice, from paper medical records. After identification of all AD patients, data were abstracted from the electronic and paper charts, and captured in a predefined electronic collection instrument. Missing data were recorded as such [8].

### Clinical suspicion

We documented the first differential diagnosis upon admission, if available. If suspicion of AD proved correct this was defined as the inclusion of this diagnosis in the first differential diagnosis. We did not differentiate between patients in whom AD was considered the most likely diagnosis, and those in whom AD was ranked second or lower. Clinical suspicion for AD was considered absent if AD was not included in the first differential diagnosis.

We compared the baseline characteristics, symptoms and signs and the applied diagnostics of both groups. Then, we identified characteristics associated with the likelihood of a correct clinical suspicion after correction for other characteristics. In the subset of patients with Stanford type A dissection, we compared the time from hospital admission to surgery in both groups.

### Patient outcomes

All-cause mortality during follow-up was registered from medical charts and by contacting the general practitioner, and was completed up to November 2013. We studied the association between clinical suspicion and long-term mortality corrected for a set of clinical variables: i. e. sex, age, hypertension, extracardiac atherosclerosis, chronic obstructive pulmonary disease, a pulse deficit, and haemoglobin on admission. Since this association has not been studied previously, variables were selected based on clinical reasoning.

### Statistical analysis

Throughout the study we compared patients with and without AD in the first differential diagnosis, unless stated otherwise. Nominal variables were presented as frequencies and percentage of total, continuous variables as the median and 25^th^ and 75^th^ percentile. Statistical difference of nominal variables was tested with a Chi-square test or Fisher’s exact test as appropriate; differences in the distribution of continuous variables were tested with a Mann-Whitney‑U test. Associates of the clinical suspicion were assessed using a multivariable log-binomial regression model, which can be interpreted as a relative risk. Survival plots were calculated with Kaplan-Meier statistics; as a reference we depicted the expected survival of age- and sex-matched Dutch inhabitants. Differences in the survival were assessed using a univariable and multivariable Cox proportional hazards model, which included the previously mentioned variables. Missing data were imputed, since discarding subjects with missing data can result in a lower statistical power and may introduce bias due to the non-random missingness of variables [9–12]. Twenty imputed datasets were created using the appropriate software in SPSS version 22.0, the results were then pooled using Rubin’s rule [13]. Figures were created in R version 2.13.1. A *p*-value < 0.05 was regarded statistically significant throughout the study.

## Results

### Study population and missing data

We identified 213 patients diagnosed with AD of whom 13 were excluded because of iatrogenic or traumatic dissection (*n* = 9), surgical treatment at a referring hospital (*n* = 3) or aged <18 years (*n* = 1). The cohort thus consisted of 200 patients, including 152 (76.0%) patients with Stanford type A AD and 10 (5.0%) patients with an intramural haematoma. Acute aortic dissection was included in the first differential diagnosis in 138 patients (69.0%), while in 62 patients (31.0%) AD was not suspected initially. Baseline characteristics were missing in ≤3.0% of patients and any component of the physical examination was missing in ≤11.5% of patients.

### Patient characteristics, symptoms and signs

The baseline characteristics of both groups are depicted in Table 1. The median age of all patients was 64 years, which was similar in both groups. AD was more often not initially suspected in women (50.1% vs. 34.0%, *p* = 0.046); other baseline variables were not statistically different.

**Table 1:** Baseline characteristics

	In first DD (*n* = 138)		Not in first DD (*n* = 62)		*P*
Age	64	(54–73)	65	(56–72)	0.74
Female sex	47	(34.0)	31	(50.1)	0.046
BMI	26	(24–29)	26	(24–29)	0.90
Current smoking	56	(40.9)	23	(36.9)	0.67
*History of:*					
Hypertension	78	(56.3)	41	(66.5)	0.22
COPD	26	(18.8)	13	(21.6)	0.66
Diabetes mellitus	9	(6.5)	5	(7.2)	0.84
Extracardiac atherosclerosis	25	(18.4)	19	(30.0)	0.09
Connective tissue disease	17	(12.3)	2	(3.0)	0.10
Myocardial infarction	14	(10.2)	7	(11.0)	0.88
Neurological dysfunction	17	(12.6)	12	(19.2)	0.24
Previous aortic dissection	7	(5.2%)	1	(1.4%)	0.99
Cardiac surgery	13	(9.6)	4	(6.8)	0.56
Bicuspid aortic valve	10	(12.3)	2	(3.4)	0.36

Table depicts the baseline characteristics of patients with and without the inclusion of acute aortic dissection (AD) in the first differential diagnosis (DD)

*BMI* body mass index, *COPD* chronic obstructive pulmonary disease

Chest pain was reported similarly in both groups (64.7% vs. 61.3%, *p* = 0.68, Table 2), as were the absence of pain (10.2% vs 18.2%, *p* = 0.15) and a sudden onset (79.4% vs. 67.9%, *p* = 0.16). Patients differed significantly, however, with respect to the presence of back pain, which was higher in the group suspected of having AD (56.1% vs. 31.0%, *p* = 0.004). Haemodynamic parameters including the heart rate, blood pressure and haemoglobin level were similar.

**Table 2:** Symptoms and signs

	In first DD (*n* = 138)		Not in first DD (*n* = 62)		*P*
Stanford type A AD	108,108	(78.3)	44	(70.9)	0.210.28
Intramural haematoma	6	(4.3)	3	(4.8)	0.85
*Symptoms*					
Chest pain	89	(64.7)	38	(61.3)	0.68
Back pain	77	(56.1)	19	(31.0)	0.004
Abdominal pain	33	(23.7)	15	(24.0)	0.98
No pain reported	14	(10.2)	11	(18.2)	0.15
Migration of pain	28	(20.0)	6	(9.5)	0.16
Sudden onset	110	(79.4)	42	(67.9)	0.10
Focal neurological deficit	22	(15.8)	6	(9.9)	0.23
TLOC	27	(19.5)	7	(10.7)	0.18
Coma	17	(12.1)	7	(10.7)	0.77
*Signs*					
Any pulse deficit	29	(20.7)	6	(9.9)	0.13
Heart rate	73	(62–90)	78	(65–90)	0.19
Systolic BP (mm Hg)	120	(95–160)	120	(105–170)	0.53
Diastolic BP (mm Hg)	67	(50–85)	70	(60–90)	0.35
Haemoglobin (mmol/l)	7.8	(6.6–8.7)	8.0	(7.5–8.9)	0.18
Creatinine (μmol/l)	104	(90–128)	102	(85–127)	0.92

Table depicts the symptoms and signs of patients with and without the inclusion of acute aortic dissection (AD) in the first differential diagnosis (DD)

*TLOC* transient loss of conscience, *BP* blood pressure

No differences were found between the symptoms and signs of both sexes (Supplement A), except for differences in baseline creatinine (108 vs. 93 μmol/l, *p* = 0.001) and haemoglobin (8.1 vs. 7.7 mmol/l, *p* = 0.009). Patients without back pain differed with respect to several other symptoms and signs (Supplement B), i. e. these patients more often had type A AD (55.7% vs. 89.9%, *p* < 0.001), ‘painless’ AD (0 vs. 25.8%, *p* < 0.001), and transient (3.5% vs. 21.3%, *p* < 0.001) or persistent loss of consciousness (1.2 vs. 11.2%, *p* = 0.006); a sudden onset was reported less often (65.9% vs. 88.6%, *p* < 0.001). Also, the median systolic blood pressure was lower (144 mm Hg vs. 113 mm Hg, *p* < 0.001) and the heart rate higher (78 bpm vs. 73 bpm, *p* = 0.03) in these patients.

### Diagnostic tests

A chest X‑ray was more often taken in patients initially not suspected of AD (69.5% vs. 84.6%, *p* = 0.04); the presence of a widened mediastinum (44.2% vs. 46.1%, *p* = 0.89) or pleural effusion (9.3% vs. 7.8%, *p* = 0.76) was similar in both groups. The electrocardiogram showed atrial fibrillation in 5.7 and 5.3% (*p* = 0.92) in the two groups, respectively; the incidence of ST depression (18.0% vs. 30.2%, *p* = 0.08) and ST elevation (9.9% vs. 15.1%, *p* = 0.33) was not significantly different between the groups. D‑dimer was measured in 16 patients, 12 of whom had AD included in the first differential diagnosis. All D‑dimer assays were positive, i. e. above the conventional threshold of 0.50 μg/ml and also above the age-adjusted cut-off (age/100 μg/ml).

Table 3 compares the primary and total number of imaging modalities in both groups. In patients not initially suspected of AD, the primary imaging test was more often transthoracic echocardiography (TTE; 34.7% vs. 50.9%) or coronary angiography (9.1% vs. 0.8%) and less often computed tomography (CT; 44.5% vs. 29.1%) or transoesophageal echocardiography (TEE; 5.5% vs. 15.1%; *p* for all categories = 0.002). The average number of imaging tests carried out was higher in patients not initially suspected of AD (1.8 vs. 2.3, *p* = 0.01). Comparing all the imaging tests carried out, TTE (52.1% vs. 70.9%, *p* = 0.02) and coronary angiography (2.5% vs. 18.2%, *p* < 0.001) were more often used in the ‘not suspected’ group, while CT (77.3% vs. 67.3%, *p* = 0.16), TEE (45.4% vs. 42.6%, *p* = 0.73) and MRI (5.0% vs. 7.3%, *p* = 0.76) were used similarly in both groups.

**Table 3:** Imaging tests

	In first DD (*n* = 138)		Not in first DD (*n* = 62)		*P*
*First imaging test*					
TTE	43	(36.1)	28	(50.9)	0.002*
CT	53	(44.5)	16	(29.1)	–
TEE	18	(15.1)	3	(5.5)	–
MRI	3	(2.5)	0	(0)	–
CAG	1	(0.8)	5	(9.1)	–
*All imaging tests*					
TTE	62	(52.1)	39	(70.9)	0.02
CT	92	(77.3)	37	(67.3)	0.16
TEE	54	(45.4)	23	(42.6)	0.73
MRI	6	(5.0)	4	(7.3)	0.56
CAG	3	(2.5)	10	(18.2)	<0.001

Table compares the first and total number of tests used in patients with and without AD in the first differential diagnosis

*P-value for all categories

*TTE* transthoracic echocardiography, *CT* computed tomography, *TEE* transoesophageal echocardiography, *MRI* magnetic resonance imaging, *CAG* coronary angiography

### Clinical suspicion

In 179 patients we were able to retrieve a differential diagnosis; in 124 (69.3%) of these patients AD was included, and in 91 (50.8%) patients it was considered the most likely diagnosis. In the 124 patients suspected of AD, this diagnosis was positioned first, second and third or higher in 73.4, 19.4, and 7.2% of patients, respectively. Corrected for other baseline characteristics, AD was less likely to be included in the first differential diagnosis in patients without back pain (RR 0.51, 95% CI: 0.30–0.84; Table 4), in female patients (RR 0.66, 95% CI: 0.44–0.99) and in patients with extracardiac atherosclerosis (RR 0.64, 95% CI: 0.43–0.96).

**Table 4:** Factors influencing the probability of the correct inclusion of an acute aortic dissection in the first differential diagnosis

	RR	(95% CI)	*P*
Female sex	0.66	0.44–0.99	0.04
Age	0.99	0.98–1.01	0.55
Extracardiac atherosclerosis	0.64	0.43–0.96	0.03
Connective tissue disease	3.17	0.49–20.7	0.23
Absence of back pain	0.51	0.30–0.84	0.008
Migratory pain	1.37	0.42–4.39	0.60

Table shows the multivariable-adjusted relative risk (RR) for the correct inclusion of an aortic dissection in the first differential diagnosis. An RR > 1 indicates that a correct suspicion of an AD is more likely, while an RR < 1 indicates a lower likelihood for the mentioning of an AD

### Patient management and outcomes

Type A AD was treated surgically in 136 of 152 (89.5%) patients. The median time to surgery was 1.8 (0.8–4.1) hours in the ‘suspected’ group versus 10.1 (2.2–26.9) hours in the ‘not-suspected’ group (*p* < 0.001). Seven patients with type A AD died before surgery could be initiated. Nine patients died during surgery for type A AD, with a similar distribution among patients with and without an initial suspicion for AD (5.6% vs. 9.4%, *p* = 0.56). In nine patients conservative treatment was chosen because of patient preference, old age or comorbidity; one of these patients survived until hospital discharge. The overall in-hospital mortality was similar in both groups (24.2% vs, 27.3; *p* = 0.66).

**Fig. 1:**
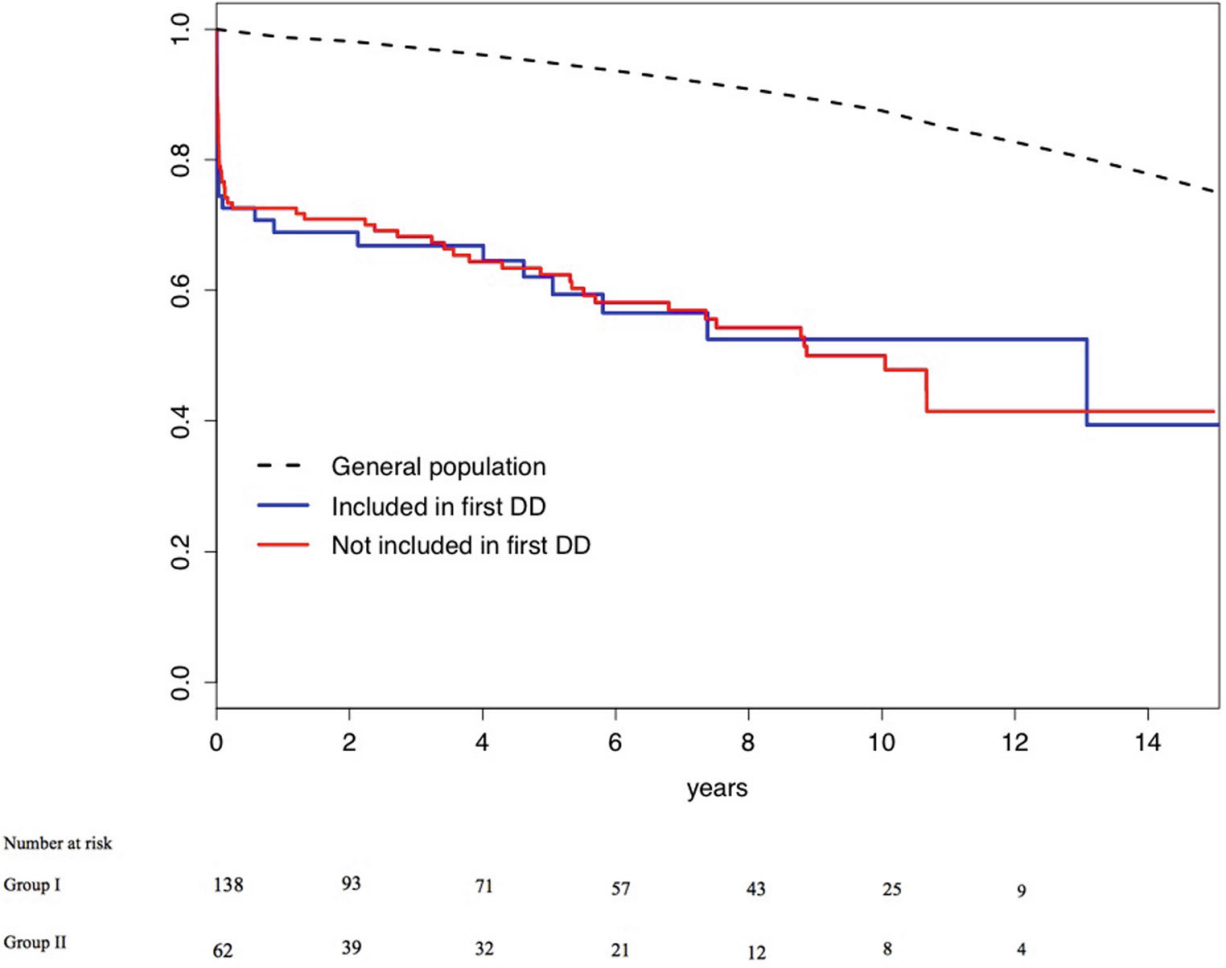
Long-term survival of patients in whom AD was included in the first differential diagnosis (Group I, *blue line*) and patients in whom AD was not included in the first differential diagnosis (Group 2, *red line*); as a reference the age- and sex matched survival of Dutch inhabitants is depicted (*black dashed line*). A *p*-value is not given because lines cross

Median follow-up of all-cause mortality was 6.3 (interquartile range: 3.3–9.4) years, during which 92 (46.0%) patients died. Thirty-day mortality was similar in both groups (23.4 and 25.5% respectively, *p* = 0.77), as was the long-term survival (Fig. 1). The hazard ratio comparing patients both with and without AD in the first differential diagnosis was 0.97 (95% CI: 0.61–1.55), and 0.76 (95% CI: 0.46–1.27) after multivariable correction.

## Discussion

This study showed that AD was not recognised during the primary clinical assessment in one-third of patients who were eventually diagnosed with AD. Although this was associated with the use of additional diagnostic tests and a prolonged time from admission to surgery, the short- and long-term outcomes were similar. Reduction of the time lapse between recognition and treatment of aortic dissections is of the utmost importance to lower perioperative mortality. We suggest that physicians who attend patients with suspected AD need to be familiar with the pitfalls in identifying symptoms and signs. Primarily, these are a low incidence combined with a broad range of symptoms which can mimic other conditions such as stroke, myocardial infarction and other abdominal pathology. Our study extends to the insights that have been gained in the past decade from larger registries such as the International Registry of Acute Aortic Dissection (IRAD) to provide adequate information regarding the symptoms and signs of AD [14, 15]. It is also important to know how many AD patients are missed and what makes these patients different from patients who are correctly identified.

In our study, AD was not initially recognised in 31% of patients. This was more likely in the absence of back pain, in female patients, and in patients with extracardiac atherosclerosis. It is important to be aware of these associations since they may improve the awareness of AD in these patients. However, it is likely that these factors are a proxy for a different clinical picture rather than the cause for a missed diagnosis. The absence of back pain as a presenting symptom was indeed associated with other differences in the clinical presentation, as these patients more often had type A AD, presented with ‘painless’ AD, or had a transient or persistent loss of consciousness, and less often reported sudden onset; also, they had a worse haemodynamic profile. It is thus likely that these underlying differences in the clinical picture are the actual reason that patients were not adequately identified as having AD. A study by Imamura et al. similarly showed that patients who presented with painless AD more often had an atypical presentation with higher incidences of haemodynamic shock and neurological symptoms [16]. Therefore, to improve the identification of AD patients, physicians should be more aware of the possibility of AD in patients with shock of unknown cause and in patients with loss of consciousness, specifically if combined with thoracic pain.

Our results did not indicate that female sex was similarly a proxy for a different clinical profile, as the distribution of symptoms and signs was similar among males and females except for the sex-dependent haemoglobin and creatinine levels. However, previous studies show that women less often have an abrupt onset of symptoms or a pulse deficit, and more often present with coma and congestive heart failure [17]. These factors are themselves associated with delayed recognition of AD [18]. Indeed, it has been shown that the time from admission to diagnosis is longer in women than in men [18]. It is thus likely that an ‘atypical’ presentation in women is recognised less well, a phenomenon that is also well described in other cardiovascular diseases [19].

Based on our results and on previously reported findings it is clear that the diagnostic process in patients with suspected AD needs to be improved. Several aspects of the diagnostic process can be considered. First, risk scores should be used to calculate the a priori risk of AD [20, 21]. Patients with a predicted high risk should have expedited imaging with CT, TEE or MRI. Second, measurement of D‑dimer should be considered in patients with a low or intermediate a priori risk [22, 23]. The use of D‑dimer was still limited during our study, but is currently an important tool to further stratify the diagnostic process. In our study none of the patients had a false negative test; previous studies showed that D‑dimer is specifically useful to exclude AD, since the likelihood of AD after a negative test (0.50 μg/ml) is only 0.3% [24].

Finally, the inadvertent administration of anticoagulant or thrombolytic therapy to AD patients who present with symptoms suggestive of myocardial or cerebral infarction may lead to acute haemorrhage and death [25–27]. The former should be suspected in patients who present with ST elevation in the inferior leads, since progression of an intimal tear is usually to the right coronary cusp. Specifically, alternating ST elevation and concomitant neurological symptoms should raise suspicion of AD. For the timely recognition of dissections mimicking stroke, routine visualisation of the aortic arch and its branching vessels during cerebral CT may be considered. Such a protocol could, at the cost of a little extra scanning time and radiation exposure, identify patients with an underlying aortic dissection.

## Limitations

Some limitations apply to our study, mainly the retrospective study design may have precluded several biases. First, despite a great effort to register all AD patients, we may have missed patients who were managed conservatively and those who were misclassified as having another condition or who died before the diagnosis was established [3]. Also, registration of baseline characteristics may have been incomplete due to the retrospective data collection. Rather than discarding missing data, we imputed missing covariates, as this has been shown to reduce bias and increase the precision of results [10, 11]. Furthermore, the study was not designed to directly capture the effect of test results on the clinical suspicion for AD. Finally, although we consider the registration of the presence versus absence of AD in the first differential diagnosis a strength of this study, it would have been informative to also know which alternative diagnoses were considered and how this affected the diagnostic strategy.

In summary, acute aortic dissection was initially not suspected in almost one-third of patients. This was more likely to occur in women, patients with extracardiac atherosclerosis or absence of back pain. Patients without AD in the first differential diagnosis underwent more imaging tests and had a longer time from admission to surgery, but a similar short- and long-term mortality. Improved recognition of patients with acute aortic dissection could lead a reduction in the number of diagnostic tests, a shorter time to therapy, and possibly improved patient outcomes.

## Caption Electronic Supplementary Material

Comparison of the symptoms and signs in both sexes

Comparision of the symptoms and signs in patients with and without back pain on admission
