# Supplementary material for: Clinical recognition of acute aortic dissections: insights from a large single-centre cohort study
Source: Neth Heart J. 2016 Nov 23;25(3):200–6. doi: 10.1007/s12471-016-0921-8 (PMC5313444; doi:10.1007/s12471-016-0921-8)
Supplement: Supplementary file 1 — Comparison of the symptoms and signs in both sexes [file 12471_2016_921_MOESM1_ESM.docx]

|  | Male  (N = 122) | | Female  (N = 78) | | P |
| --- | --- | --- | --- | --- | --- |
| Stanford Type A | 94 | (77.0) | 58 | (74.4) | 0.66 |
|  |  |  |  |  |  |
| **Symptoms** |  |  |  |  |  |
| Any pain reported | 107 | (87.9) | 68 | (86.6) | 0.80 |
| Chest | 80 | (65.4) | 48 | (61.0) | 0.55 |
| Back | 57 | (47.0) | 39 | (50.5) | 0.65 |
| Abdominal | 24 | (19.4) | 23 | (30.4) | 0.10 |
| Migration | 16 | (13.1) | 17 | (21.9) | 0.14 |
| Sudden onset | 96 | (78.5) | 56 | (71.9) | 0.31 |
| Focal neurological deficit | 18 | (14.5) | 5 | (6.8) | 0.11 |
| TLOC | 19 | (15.6) | 14 | (18.2) | 0.66 |
| Coma | 15 | (12.6) | 8 | (9.7) | 0.56 |
| **Signs** |  |  |  |  |  |
| Any pulse deficit | 19 | (15.6) | 14 | (18.2) | 0.72 |
| Heart rate | 76 | (62 – 94) | 72 | (65 – 88) | 0.37 |
| Systolic BP (mmHg) | 120 | (104 – 155) | 120 | (80 – 170) | 0.66 |
| Diastolic BP (mmHg) | 70 | (57 – 85) | 65 | (50 – 90) | 0.31 |
| Haemoglobin (mmol/l) | 8.1 | (7.1 – 8.9) | 7.7 | (6.5 – 8.4) | 0.009 |
| Creatinine (μmol/l) | 108 | (95 – 134) | 93 | (80 – 111) | 0.001 |

**Supplement A.** Comparison of the symptoms and signs in both sexes

*TLOC* transient loss of conscience, *BP* blood pressure.
