# Supplementary material for: Clinical recognition of acute aortic dissections: insights from a large single-centre cohort study
Source: Neth Heart J. 2016 Nov 23;25(3):200–6. doi: 10.1007/s12471-016-0921-8 (PMC5313444; doi:10.1007/s12471-016-0921-8)
Supplement: Supplementary file 2 — Comparision of the symptoms and signs in patients with and without back pain on admission [file 12471_2016_921_MOESM2_ESM.docx]

|  | Back pain  (N = 88) | | No back pain  (N = 89) | | P |
| --- | --- | --- | --- | --- | --- |
| Stanford Type A | 49 | (55.7) | 80 | (89.9) | <0.001 |
| **Symptoms** |  |  |  |  |  |
| Chest | 50 | (56.8) | 61 | (68.5) | 0.11 |
| Abdominal | 24 | (27.3) | 14 | (15.7) | 0.06 |
| Migration | 18 | (20.5) | 3 | (3.4) | 0.001 |
| Painless dissection | 0 | (0) | 23 | (25.8) | <0.001 |
| Sudden onset | 78 | (88.6) | 58 | (65.9) | <0.001 |
| Focal neurological deficit | 9 | (10.5) | 10 | (11.2) | 0.87 |
| TLOC | 3 | (3.5) | 19 | (21.3) | <0.001 |
| Coma | 1 | (1.2) | 10 | (11.2) | 0.006 |
| **Signs** |  |  |  |  |  |
| Any pulse deficit | 11 | (12.9) | 11 | (12.8) | 0.98 |
| Heart rate | 73 | (60 – 85) | 78 | (65 – 100) | 0.03 |
| Systolic BP (mmHg) | 144 | (118 – 176) | 113 | (90 – 140) | <0.001 |
| Diastolic BP (mmHg) | 80 | (61 – 97) | 63 | (50 – 80) | <0.001 |
| Haemoglobin (mmol/l) | 8.0 | (7.3 – 8.9) | 7.8 | (7.3 – 8.7) | 0.39 |
| Creatinine (μmol/l) | 73 | (60 – 85) | 78 | (65 – 100) | 0.44 |

**Supplement B** Comparision of the symptoms and signs in patients with and without back pain on admission

TLOC = transient loss of conscience, BP = blood pressure.
